# CRISPR-Cas9 targeting the *bla*_KPC_ gene in a clinical isolate of *Klebsiella michiganensis*: Reduction of imipenem resistance and changes in genomic carbapenem resistance determinants

**DOI:** 10.1371/journal.pone.0328521

**Published:** 2025-08-12

**Authors:** Thaysa Leite Tagliaferri, Alex Krüttgen, Tiago Antônio de Oliveira Mendes, Simone Gonçalves dos Santos, Hans-Peter Horz

**Affiliations:** 1 Institute of Medical Microbiology, RWTH Aachen University Hospital, Aachen, Germany; 2 Department of Microbiology, Institute of Biological Sciences, Universidade Federal de Minas Gerais, Belo Horizonte, Brazil; 3 Laboratory Diagnostic Center, RWTH Aachen University Hospital, Aachen, Germany; 4 Department of Biochemistry and Molecular Biology, Universidade Federal de Viçosa, Viçosa, Brazil; Cornell University, UNITED STATES OF AMERICA

## Abstract

The CRISPR-Cas technology can be used to disable drug resistance genes. Since carbapenem resistance can be mediated by multiple resistance determinants, we here investigated the extent of re-sensizitation when targeting the *bla*_KPC_ carbapenemase gene and assessed possible effects on porins and efflux pumps. While full re-sensitization was achieved in a laboratory strain of *Escherichia coli* solely equipped with *bla*_KPC_, resistance reduction in a clinical isolate of *Klebsiella michiganensis* was achieved in 63% of analyzed transformants, which was a consequence of plasmid copy number reduction and decreased *bla*_KPC_ gene expression. Damages in the Cas9, as well as alterations in carbapenem-resistance promoting genes including *ompK36* downregulation and mutations in the *acrB* gene were found, likely preventing more efficient re-sensitization. Hence, interference with a single resistance gene promoted the emergence of clonal variants that exhibit alterations in outer membrane proteins. Those bacterial countermeasures, however, were not sufficient to restore the original carbapenem-resistant phenotype.

## Introduction

Bacterial resistance against carbapenems constitutes a major public health concern, as carbapenems are broad-spectrum antibiotics widely used for treating infections caused by multi-drug resistant (MDR) gram-negative bacteria [1]. Multiple factors are responsible for the promotion of carbapenems resistance, including porins, efflux pumps and carbapenemases [2]. The *bla*_KPC_ carbapenemase gene is one of the main cause of resistance to carbapenems and has spread worldwide [3] since its first description in *K. pneumoniae* [4]. So far, more than 140 variants of the *bla*_KPC_ gene have been described [5]. Of concern, those genes are prone to mutations during antibiotic therapy, ultimately leading to treatment failure [5]. *Klebsiella michiganensis* has been recently identified as an important carrier of *bla*_KPC_ genes, and this species has been reported as a newly emerging human pathogen [6,7]. While efforts have been made for the discovery of novel antibiotics to treat infections caused by carbapenems-resistant pathogens, alternative strategies should be explored to help combating the perpetual evolution of carbapenemase genes, as well as their dissemination [8].

One novel experimental intervention to counteract the prevalence and spread of antibiotic resistance involves the use of the CRISPR-Cas technology [9–13]. The results of experimental studies in recent years have shown the effective use of CRISPR-Cas systems as a programmed tool against antimicrobial resistant bacteria [9–13]. While some research groups explored the targeted killing of MDR bacteria [9,13], others used CRISPR-Cas to selectively eliminate plasmids harbouring resistance determinants from clinically relevant bacteria [10–12]. However, bacteria can counteract CRISPR-Cas9 activity by means of CRISPR-Cas9 system disruption [9,10,12]. In addition, bacteria might possess overlapping resistance mechanisms that can contribute to the persistent ineffectiveness of an antibiotic. Resistance reversal could therefore be challenging when CRISPR-Cas9 targets only a single resistance gene.

Here, we investigated the extent of re-sensitization when CRISPR-Cas9 targets the *bla*_*KPC*_ gene in a clinical isolate of *Klebsiella michiganensis*, bearing in mind that beyond carbapenemases, other resistance determinants such as porins and efflux pumps [2] might reduce the re-sensitization success. We therefore also analysed the response of those resistant determinants at the genomic and gene expression levels upon CRISPR-Cas9 treatment.

## Results and discussion

Initially, the gRNA sequence of the CRISPR-Cas9 system was designed to target a conserved region of the *bla*_KPC_ gene located before its catalytic site (S1A Fig). Different variants of the *bla*_KPC_ gene harboured by members of the *Enterobacteriaceae* family were selected for the gRNA design, i.e. *bla*_KPC-1_, *bla*_KPC-2_, *bla*_KPC-3_, *bla*_KPC-12_, *bla*_KPC-14_, *bla*_KPC-19_, and *bla*_KPC-25_. As a conserved region close to the 5’-end of the *bla*_KPC-2_ gene, the sequence ACCATTCGCTAAACTCGAAC was selected (S1B, C Fig).

**Fig 1:**
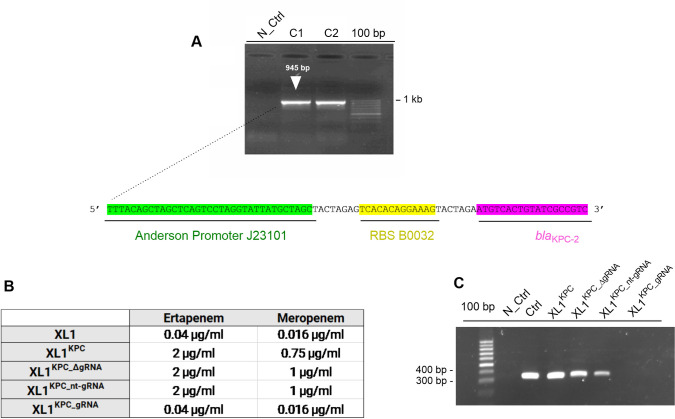
CRISPR-Cas9 restores carbapenem sensitivity in *E. coli* XL1-blue. 1A. Agarose gel showing the PCR-amplified *bla*_KPC_ gene of two clones (C1, C2) with the expected full size of 945 bp, using a forward primer (pink) containing also the Anderson promoter J23101 (green) and RBS (yellow) regions at the 5’-end, both required for *bla*_KPC_ expression. 1B. Minimal inhibitory concentrations (MIC) of ertapenem and meropenem against *E. coli* lacking the *bla*_KPC_ gene (XL1), when transformed with the *bla*_KPC_ gene (referred to as XL1^KPC^) and when XL1^KPC^ was treated with CRISPR-Cas9 without gRNA (XL1^KPC_ΔgRNA^), with the non-targeting gRNA (XL1^KPC_nt-gRNA^), and with the targeting gRNA (XL1^KPC_gRNA^). The MICs show that the antibiotic sensitivity of XL1^KPC_gRNA^ was restored to the same level as the XL1 strain. 1C. Agarose gel showing successful PCR amplification of an inner fragment of the *bla*_KPC_ gene except for XL1^KPC_gRNA^, suggesting effective re-sensitization due to *bla*_KPC_ plasmid clearance. N_Ctrl: PCR negative control; Ctrl: PCR positive control.

For verification of the gRNA-Cas9 functionality, an expression system for the *bla*_KPC-2_ gene (Fig 1A) was constructed in *E. coli* to serve as the CRISPR-Cas9 target. The generated *bla*_KPC-2_ high-copy plasmid (pUC ori, named as pTKPC) was inserted into the laboratory strain of *E. coli* XL1-blue (referred to as XL1). After introduction of the *bla*_KPC_ plasmid into XL1, the transformed strain (named as XL1^KPC^) showed increased levels of carbapenem resistance, reaching a 50-fold increase to ertapenem and a ~ 46-fold increase to meropenem when compared to XL1 (Fig 1B).

Next, XL1^KPC^ was subjected to the CRISPR-Cas9 treatment. Besides treatment with the CRISPR-Cas9 targeting the *bla*_KPC_ gene (designated as XL1^KPC_gRNA^), control experiments were performed with CRISPR-Cas9 without gRNA (designated as XL1^KPC_ΔgRNA^) and with a non-targeting gRNA (designated as XL1^KPC_nt-gRNA^). As anticipated, minimum inhibitory concentrations assays using e-tests showed that CRISPR-Cas9-based re-sensitization was attained for XL1^KPC_gRNA^, but not for XL1^KPC_ΔgRNA^ and XL1^KPC_nt-gRNA^. As a result, the original MICs observed in XL1 were restored in the XL1^KPC_gRNA^ strain for ertapenem and meropenem (Fig 1B). This re-sensitization was a consequence of plasmid clearance, evident by the lack of an amplifiable fragment of the *bla*_KPC_ gene (Fig 1C). Thus, the gRNA-Cas9 system successfully depleted the high-copy plasmid pTKPC containing the *bla*_KPC_ gene in an *E. coli* laboratory strain.

Our next goal was to test the potential of the designed gRNA-Cas9 system in reducing carbapenem resistance levels in a selected clinical isolate of *K. michiganensis* (designated as KM), considering that multiple genetic determinants may interfere with the re-sensitization efforts. Genome analysis revealed that this isolate harbours an IncN plasmid known to have a broad host range and high conjugation frequency [14]. This plasmid carries multiple resistance genes, including *bla*_KPC-2_ as sole carbapenemase gene (S2A Fig). The plasmid shows high nucleotide similarity with 756 globally spread plasmids of different bacterial species, including 350 members from the *Klebsiella* genus, and 220 members from the *Escherichia* genus (S2B Fig) [15].

**Fig 2:**
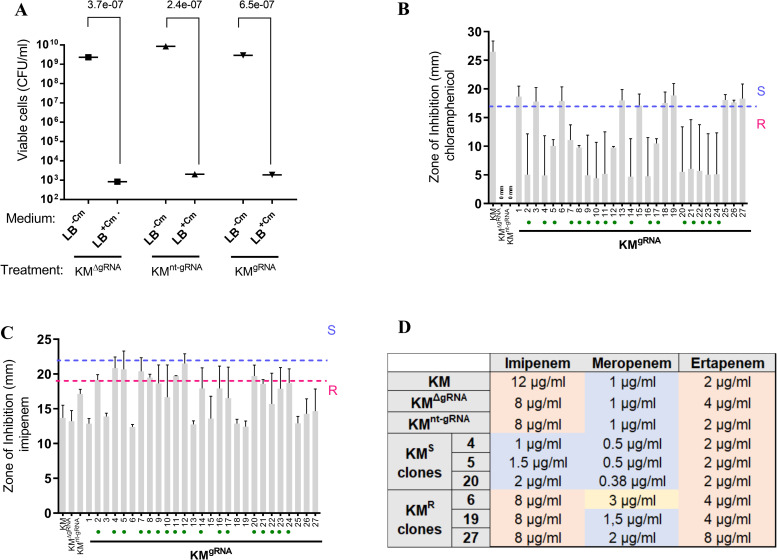
Treatment of carbapenem-resistant *K. michiganensis* (KM) with CRISPR-Cas9. 2A. Ratio of viable cells (CFU/mL) grown on solid LB medium with and without addition of chloramphenicol, designated as LB^+Cm^ and LB^-Cm,^ respectively. 2B,C. Zones of inhibition of chloramphenicol and imipenem against transformed clones determined by disk diffusion tests. Most transformants (i.e., 17/27, green dots) obtained the intended chloramphenicol resistance and showed reduced resistance against imipenem. Error bars represent standard deviation of duplicate measurements of the disk diffusion assays. 2D. Minimal inhibitory concentrations (MICs) for selected transformants confirm imipenem re-sensitization, and a two-fold reduction of meropenem sensitivity, while ertapenem sensitivity was not affected. Numbers indicate the transformant ID, as given in 2B and 2C. Colours indicate resistance phenotype (red), intermediate susceptibility (yellow) and sensitivity (blue). KM^ΔgRNA^: KM treated with CRISPR-Cas9 without gRNA; KM^nt-gRNA^: KM treated with CRISPR-Cas9 with a non-targeting gRNA; KM^gRNA^: KM treated with CRISPR-Cas9 with the targeting gRNA. KM^S^: imipenem re-sensitized transformants of KM^gRNA^; KM^R^: transformants of KM^gRNA^ with continued imipenem resistance.

Upon treatment of *K. michiganensis* with the targeting CRISPR-Cas9 (designated as ‘KM^gRNA^’), along with the controls without gRNA (KM^ΔgRNA^) and with a non-targeting gRNA (KM^nt-gRNA^), a comparably high proportion of transformants was recovered on solid media containing chloramphenicol (Cm) as selective marker for all CRISPR-Cas9 treatments (Fig 2A). This suggests that the selected targeting gRNA sequence might not be cytotoxic for *K. michiganensis*.

The antibiotic susceptibility of all retrieved 27 transformants of KM^gRNA^ grown on Cm plates was further investigated with the disk diffusion assay. While 10/27 transformants were sensitive to Cm with maintained resistance against imipenem (collectively designated as KM^R^), 17/27 transformants obtained the intended resistance against Cm and showed increased sensitivity levels to imipenem (collectively designated as KM^S^, highlighted with green dots in Fig 2B, C). This high correlation between inverse Cm and imipenem resistance phenotypes (r = 1.0, P < 0.0001, n = 27) is attributable to the introduction and performance of the pSB1C3 plasmid and CRISPR-Cas9 system in the clones. Re-sensitization success towards imipenem for selected clones could further be confirmed by determination of the MIC (Fig 2D). However, while an around two-fold increase of sensitivity was achieved for meropenem, no resistance reduction was observed for ertapenem (Fig 2D). This difference in carbapenem susceptibilities suggests a higher hydrolysis activity of *bla*_KPC-2_ against imipenem, which is in line with previous studies where *bla*_KPC-2_ activity was compared with other gene variants such as *bla*_KPC-4_ and *bla*_KPC-5_ [16] or even among sub-variants of the *bla*_KPC-2_, such as *bla*_KPC-2C_ [17].

**Fig 3:**
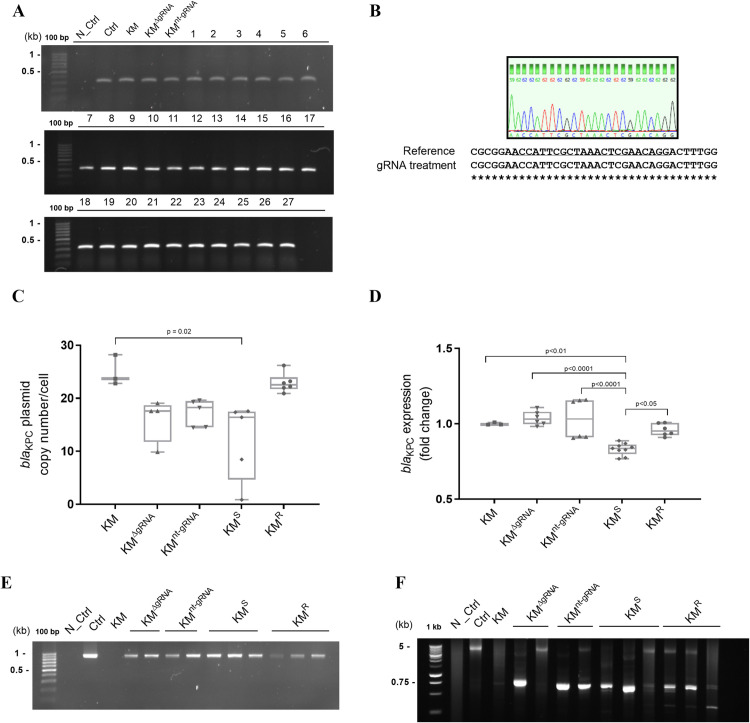
Genetic analysis of CRISPR-Cas9 mediated re-sensitization of *K. michiganensis* (KM). 3A. Agarose gel showing PCR amplification of a *bla*_KPC_ gene fragment in all transformants indicating that re-sensitization was not due to *bla*_KPC_ plasmid clearance. 3B. Sanger sequencing of representative transformants shows that the gRNA targeting region of the *bla*_KPC_ gene is intact. 3C. Copy number of the *bla*_KPC_ plasmid determined via real-time quantitative PCR. A significant reduction was observed in the re-sensitized cells KM^S^. 3D. Fold change *bla*_KPC_ gene expression. A significant reduction was observed in the re-sensitized cells KM^S^ likely due to a decrease in *bla*_KPC_ gene copy number, as seen in 3C. 3E. Agarose gel showing PCR amplification of a fragment of the CRISPR-Cas9 carrying vector indicating its successful transformation in all assessed transformants. 3F. Agarose gel showing PCR amplification of the entire CRISPR-Cas9 system (~ 5.4 kb), indicating compromised integrity in representative transformants. N_Ctrl: PCR negative control; Ctrl: PCR positive control. For further information regarding abbreviations, see legend of Fig 2. Statistical analysis was performed using one-way ANOVA.

The achieved sensitivity to imipenem was not due to a complete CRISPR-Cas9 mediated plasmid clearance, as evident by positive PCR amplification of the *bla*_KPC_ gene in all transformants (Fig 3A). Sanger sequencing revealed even a full integrity of the *bla*_KPC_ sequence region targeted by the gRNA (Fig 3B), which was later confirmed by whole genome sequencing. Therefore, it was deemed possible that imipenem re-sensitization was due to a reduction of the *bla*_KPC-_plasmid copy numbers and/or reduction in *bla*_KPC_ gene expression. In fact, a significant reduction of plasmid copy numbers was observed in the KM^S^ group but not in the KM^R^ group compared to the untreated control (KM) (Fig 3C). However, reduction in *bla*_KPC_-plasmid copy numbers was also observed in the controls KM^ΔgRNA^ and KM^nt-gRNA^, possibly the result of a detrimental effect that can occur during plasmid co-infection [18]. Importantly, the *bla*_KPC_ gene expression was significantly reduced in the KM^S^ transformants compared to all other groups (Fig 3D). Hence, re-sensitization to imipenem was a result of plasmid copy number reduction and decreased *bla*_KPC_ gene expression. Further studies are required to investigate the stability of such re-sensitization.

As the *bla*_KPC_ plasmid was not completely cleared from the cells, the CRISPR-Cas9 system could have been impaired. Therefore, two different PCR reactions were performed, confirming on the one hand the presence of the CRISPR-Cas9 plasmid in the cells (Fig 3E), and on the other hand revealing deletions within the CRISPR-Cas9 locus (~5.4 kb) in all treatment groups, except for the control plasmid Ctrl (Fig 3F). Mutations in the CRISPR-Cas9 locus have been previously described and have for instance been linked to the presence of toxin-antitoxin genes in the targeted plasmid [9,10,12]. However, no such genes have been detected in the *bla*_KPC_ plasmid *of K. michiganensis* in our study. Instead, it is possible that the bacterial cells benefit from those mutations, even in the controls, due to reduced cell burden caused by the Cas9, which can alter cell behaviour even without the DNA cleavage activity (dCas9) and a targeting gRNA [19].

**Fig 4:**
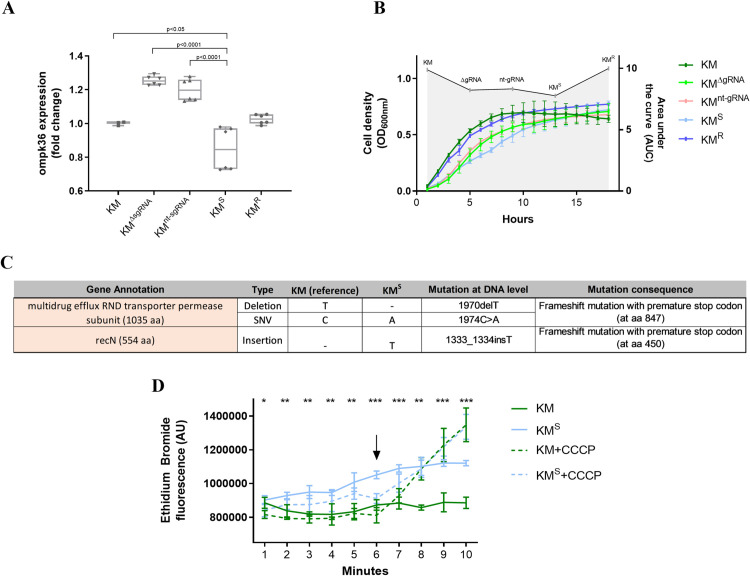
Changes in two transmembrane proteins involved in carbapenem resistance upon CRISPR-Cas9 treatment. 4A. Relative expression of *ompK36* gene was significantly downregulated in the re-sensitized transformants (KM^S^) compared to the controls. Dots represent the expression values of the individual transformants tested. 4B. Bacterial growth curves of KM with or without previous CRISPR-Cas9 treatment determined by optical density measurements (OD_600nm_). Significantly reduced fitness was observed in the re-sensitized transformants (KM^S^). Significance compared to KM was given from timepoints 2 hours to 11 hours; significance compared to KM^ΔgRNA^ and KM^nt-gRNA^ controls was given from timepoints 6 hours to 8 hours. 4C. Genetic alterations identified by whole genome sequencing in a representative KM^S^ transformant*.* A frameshift mutation was identified in the *acrB* gene along with one nucleotide exchange further downstream. Additionally, a frameshift mutation was detected in the *recN* gene. 4D. Putative damages in the efflux pump AcrAB-TolC as indicated by WGS were confirmed by significantly higher accumulation of ethidium bromide in three biological replicates of KM^S^ transformants when compared to KM (blue and green solid lines). Differences in the ethidium bromide accumulation were abolished (blue and green dashed lines) when an artificial efflux pump inhibitor (CCCP) was added at the 6^th^ minute, indicated by an arrow. P-values refer to significant differences between KM and KM^S^ at each displayed time point (without addition of CCCP, solid lines). *p < 0.05; **p < 0.01; ***p < 0.001. Statistical analysis was performed using one-way ANOVA.

Besides carbapenemases, other known intrinsic carbapenem resistance mechanisms in *Klebsiella* are the outer membrane porin OmpK36, as well as the multidrug efflux RND transporter [2,17,20,21]. Therefore, we assessed whether the CRISPR-Cas9 treatment targeting the *bla*_KPC_ gene led to mutational changes and expression levels in the above transporters. While whole genome sequencing of one KM^S^ transformant revealed no mutation in the *ompK*36 gene, the expression of this gene was significantly downregulated in the KM^S^ group (Fig 4A). As porins are also responsible for nutrient uptake [22,23], we measured bacterial growth fitness. Fig 4B shows that the KM^S^ transformants have the lowest fitness during the exponential growth phase, being significantly different from all other groups. This is likely in agreement with the reduced *ompK*36 gene expression, which may lead to an important competitive disadvantage compared to bacterial cells with a fully expressed OmpK36 [23,24].

Apart from changes in the *ompK36* expression, whole genome variant analyses of one representative KM^S^ transformant revealed a mutation in the multidrug efflux RND transporter permease subunit. Annotation with prokka and Pfam analysis of the protein sequence linked this subunit to the Acriflavine resistance (acrB) gene. The gene has a single nucleotide deletion (1970delT), leading to a frameshift mutation and a premature stop codon, as well as one single nucleotide variant (1974C > A) (Fig 4C). However, additional replicates of genome sequencing are needed to clearly link the observed mutations to the KM^S^ phenotype. The overall reduced activity of efflux pumps in KM^S^ compared to KM could be independently confirmed by the unequal EtBr accumulation inside the bacterial cells (Fig 4D). Such disparities between EtBr accumulation were abolished when the efflux pump inhibitor CCCP was added to the bacterial culture.

AcrB is part of the AcrAB-TolC efflux complex and is responsible for substrate specificity and for export efficiency [25]. Meropenem and ertapenem (both with a lipophilic side chain, unlike imipenem) are likely substrates of the AcrB efflux pump [26]. Inactivation of genes encoding components of the AcrAB-TolC efflux pumps or efflux inhibition has been linked to increased carbapenem resistance in *Enterobacteriaceae* species [21]. In *E. coli,* carbapenem-resistant lineages also developed mutations in the *acrB* gene, including premature stop codons and a likely truncated version of AcrB [27]. The observed changes of the porins and efflux pumps could therefore be viewed as bacterial countermeasures to compensate for the CRISPR-Cas9 intervention with the *bla*_KPC_ gene. However, if so, they were not efficient enough to restore the original imipenem resistance levels.

An additional mutation in KM^S^ was observed in the *recN* gene (1333_1334insT). RecN is part of the SOS response to DNA damages being involved in the double-strand break repair pathway [28]. Additionally, the SOS response can also contribute to resistance evolution [29] and a possible link between this mutation and the CRISPR-Cas9 treatment are of interest and could be investigated in further studies.

## Conclusion

In summary, re-sensitization to imipenem could be achieved by CRISPR-Cas9 targeting the *bla*_KPC-2_ gene in a clinical isolate of *K. michiganensis* despite downregulation of gene expression and mutations affecting the carbapenem resistant determinants OmpK36 porins and AcrAB-TolC efflux pump, respectively. When the goal of tailored CRISPR-Cas9 systems is plasmid removal rather than killing antibiotic-resistant bacteria, RNA transcription profiling may be necessary to better understand the holistic and multi-faceted impact of CRISPR-Cas9 on the recipient cells. Additionally, it may help elucidate the participation of complementary and non-targeted resistance mechanisms upon CRISPR-Cas9 re-sensitization treatments. This is particularly important given that the intrinsic CRISPR-Cas9 seems to also modulate virulence genes involved in bacterial adherence, biofilm formation, antimicrobial resistance and cell mobility [30]. The identification of further, yet unanticipated genes in the maintenance of antibiotic resistance levels could improve future interventions efforts via CRISPR-Cas9 by accounting for all multiple resistance determinants which collectively confer an antibiotic-resistant phenotype.

## Methods

A general overview of the experimental design and workflow is displayed in S1D Fig.

### Bacterial strains and cultivation

Bacteria were cultivated overnight in LB medium or agar at 37 °C (unless otherwise stated) and supplemented with ampicillin 100 μg/ml or chloramphenicol (Cm) 30 μg/ml whenever necessary. The carbapenem-sensitive laboratory strain *Escherichia coli* XL1-blue was selected for this study.

In order to make *E. coli* XL1-blue strain carbapenem resistant, the *bla*_KPC-2_ gene was amplified with specific primers designed for this study (S1 Table). The forward primer contained a gene expression system (constitutive promoter, ribosome binding site – RBS) in the 5’-end, adjacent to the initial ATG of the *bla*_KPC_. The J23101 selected promoter has a higher expression level compared to other promoters from the same family and the RBS, named K1725317, has a medium estimated strength [31]. This set of primers enabled the PCR cloning and expression of the *bla*_KPC_ gene into the 2.1-TOPO vector (pUC origin) (Thermo Fischer Scientific, MA, United States) which lacks its own expression cassette. The *E. coli* strain XL1-blue was made chemically competent and the *bla*_KPC_ plasmid was introduced via heat-shock [32].

*K. michiganensis* was isolated from a clinical specimen at the University Hospital RWTH Aachen, Germany. Its antibiotic resistance profile was determined using the VITEK2 system (bioMérieux, France). The presence of carbapenem resistance genes was first screened using the Xpert Carba-R kit (Cepheid, USA) and confirmed by analysis of the bacterial genome via ResFinder [33].

### Disk diffusion test and minimum inhibitory concentration

Antibiotic resistance levels of bacterial isolates were evaluated using either disk diffusion or minimum inhibitory concentration (MIC) tests. The disk diffusion test was performed on Mueller-Hinton agar according to the Clinical and Laboratory Standards Institute (CLSI) protocol [34]. The antibiotics used were Ertapenem (10 µg), Meropenem (10 µg), Imipenem (10 µg) and Cm (30 µg). The MIC of *K. michiganensis* was evaluated using e-test strips (bioMérieux, France) according to the manufacturer’s instructions. Disk diffusion and MIC resistance levels were interpreted according to EUCAST v 14.0 (2024).

### CRISPR-Cas9 plasmid description and gRNA design

The pSB1C3 plasmid used in this study contains a constitutively expressed CRISPR-Cas9 system derived from *Streptococcus pyogenes*. The gRNA targeting the *bla*_KPC_ gene was designed by indoor scripts using Pearl language based on public available sequences of the 882 bp-long *bla*_KPC_ gene and its variants *bla*_*KPC-1*_*, bla*_*KPC-2*_*, bla*_*KPC-3*_*, bla*_*KPC-12*_*, bla*_*KPC-14,*_ *bla*_*KPC-19*_*, and bla*_*KPC-25*_ (S1 Fig). The sliding window method was used to find all 20-nucleotide length sequences with NGG flanking the 3-prime end. Of 293 sequences fulfilling the principal gRNA requirements of Cas9, 111 sequences were conserved across the analysed variants. The gRNA sequence (ACCATTCGCTAAACTCGAAC) was carefully selected to target a conserved region upstream to the active site of the KPC enzyme, placed in the amino acid position 69 (S1 Fig). This way, in case of an eventual bacterial DNA repair mechanism, we intended to maximize the likelihood of generation of an early stop codon leading to enzyme disruption. Potential off-target regions were investigated using the Cas-OFFinder programme [35]. After synthesis of the gRNA sequence, it was phosphorylated using a polynucleotide kinase (Thermo Fischer Scientific, MA, United States) and ligated into the plasmid pSB1C3 between the CRISPR repeats. Ligation success was confirmed by Sanger sequencing (S1 Fig). For the non-targeting gRNA design, a conserved sequence within the green fluorescent protein (GFP, BBa_E0040 [31]) gene was designed (GGGCACAAATTTTCTGTCAG) as described above. The pSB1C3 plasmid was transformed into the targeted cells via electroporation and successful transformants were retrieved from plates supplemented with Cm (resistance marker of pSB1C3), to which the cells were previously sensitive. The degree of Cm resistance acquisition was then evaluated by disk diffusion, as described above.

### Polymerase chain reactions (PCR)

The primers used for PCR and the reaction conditions are displayed in supplementary S1 Table. The copy number of the *bla*_KPC_ carrying plasmid was determined using quantitative PCR. To this end, amplification products of the 16S rRNA gene and the *bla*_KPC_ gene were cloned into the TOPO vector (Thermo Fischer Scientific, United States) and used as subsequent standards for quantitative PCR [36]. Absolute bacterial cell numbers were determined based on the average 16S rRNA gene copy number per cell [37,38].

The expression of the *bla*_KPC_ and *ompK*36 genes was assessed by quantitative reverse transcription PCR (RT-qPCR) of total RNA. The 16S rRNA was used as endogenous transcript for the internal control. Total bacterial RNA was extracted using TRIzol and DNA was removed using DNase I (both Thermo Fischer Scientific, United States). One μg of total RNA was used for reverse transcription according to the iScript cDNA Synthesis Kit protocol (Bio-Rad, United States). RT-qPCR was performed using the SYBR Green Master Mix (Bio-Rad, United States). The analyses were performed based on the relative standard curve method using a serial dilution of total cDNA pool [39].

### Sequencing and bioinformatic analyses

Sanger sequencing was performed by Eurofins Genomics (Luxembourg) and analysed using the SeqTrace software [40]. Deep sequencing of the genomes of *K. michiganensis* was performed via the Miseq platform (Illumina, United States) according to the protocol Nextera XT DNA Sample Preparation Guide. The MiSeq v2 Reagent Kit was used to generate the 2x 150 bp reads. Read quality was assessed by FastQC [41] and the Trimmomatic [42] program was used to trim the reads to Phred15. The de novo assembly of the contigs was performed using SPAdes [43]. Annotation was performed using Prokka [44]. Resistance genes were detected via ResFinder [33]. *K. michiganensis* plasmid was characterized using the PLSDB database [45] by comparison against the plasmid records to calculate their similarity using the Mash’s distance estimation approach (accessed on 14.02.2024, Mash distance strategy, p-value 0.1; max distance 0.1). Genetic variant analyses were performed using the Phread15 trimmed reads as templates via the CLC Genomics Workbench (Qiagen, Germany). The presence of toxin-antitoxin systems was investigated using the TASmania database search [46].

### Growth curves and efflux measurement

For the growth curves, overnight cultures of bacteria were adjusted to an OD_600nm_ of 0.1 and re-grown in LB medium. The growth kinetics were recorded for 18 h at 37 °C using the microplate reader SpectraMax I3 (Molecular Devices, Sunnyvale, United States of America). The OD_600nm_ was measured at time intervals of 20 min for the entire duration of the experiment.

Efflux activity was evaluated via accumulation of ethidium bromide into the bacterial cells [47]. Overnight cultures of *K. michiganensis* were refreshed, followed by centrifugation and pellet resuspension in 100 ml of 0.05 M of Na2PO4 buffer, pH 7.0, containing 100 mM NaCl. Cells were again centrifuged and resuspended in 0.05 MNa2PO4, buffer pH 7.0, containing 100 mM NaCl and 50 mM sodium formate. Cells were then adjusted to an OD_600nm_ of 0.5. Ethidium bromide accumulation into the cells was measured by adding 180 µl of adjusted cells to each well, as well as 20 µl of 100 µM ethidium bromide. The microtiter plates were placed into the microplate reader SpectraMax i3 (Molecular Devices, Sunnyvale, U.S.) for fluorescence measurement (excitation: 530 nm, emission: 600 nm). Measurements were recorded every minute for five minutes. After this time, the efflux activity was artificially inhibited by adding 50 µM CCCP (carbonyl cyanide *m*-chlorophenylhydrazone; Sigma Chemical) to each cell sample and the measurement was continued until a total of ten minutes.

### Statistical analysis

Significant differences between the treatment groups were assessed using one-way ANOVA after confirming data normality. Correlation between inverse Cm and imipenem resistance phenotypes was assessed via Spearman’s rank correlation. Additionally, the area under the curve calculations (AUC) were performed with GraphPad Prism version 8. Graphic visualizations were also made using GraphPad Prism v8.

## Supporting information

S1 Fig Experimental design. S1A. Full *bla*_KPC_ gene sequence and the position of the designed gRNA at the 5’ end, before the region encoding for the active site of the KPC S1B. Alignment of representative *bla*_KPC_ variants from Genbank showing that the designed gRNA targets a conserved region. The selected genes and sequence accession number are: 1. *bla*_KPC-2_ KT001101.1; 2. *bla*_KPC-25_ KU216748.1; 3. *bla*_KPC-2_ KT001098.1; 4. *bla*_KPC-2_ KT001097.1; 5. *bla*_KPC-19_ KJ775801.1; 6. *bla*_KPC-3_ AF395881.1; 7*. bla*_KPC-1_ AF297554.1; 8*. bla*_KPC-12_ HQ641421.1; 9. *bla*_KPC-2_ GU086225.1; 10 *bla*_KPC-14_ JX524191.1. S1C. Sequence fragment of the CRISPR-Cas9 plasmid showing correct insertion of the gRNA. Green line below the assigned nucleotides indicates sequencing quality, according to the colour code legend. S1D. Schematic workflow of the experimental study design. Briefly, the three CRISPR-Cas9 plasmids used in this study were first constructed. CRISPR-Cas9 mediated re-sensitization ability was validated in a laboratory strain of *E. coli*. Following successful re-sensitization, the CRISPR-Cas9 effect was evaluated on a clinical strain of *K. michiganensis*.
(TIF)

S2 Fig Resistance profile and *bla*_KPC_ plasmid characterization. S2A. Resistance profile of *K. michiganensis* KM and equivalent resistance genes determinants identified by WGS. S2B. The *bla*_KPC_ gene is carried by an IncN plasmid, which has nucleotide similarity with plasmids mostly harboured by Enterobacteriaceae, especially *Klebsiella* and *Escherichia coli*. PLSDB database graphic: Centred circle “Ent…”: Enterobacteriaceae; “Ent…”: *Enterobacter*; “Citr…”: *Citrobacter*; “Sal…”: *Salmonella*; “unk”: unknown.
(TIF)

S3 Fig Original electrophoresis gel images. (PDF)

S1 Table PCR primers and conditions. (XLSX)
