# Supplementary figures and images for: CRISPR-Cas9 targeting the blaKPC gene in a clinical isolate of Klebsiella michiganensis: Reduction of imipenem resistance and changes in genomic carbapenem resistance determinants
Source: PLoS One. 2025 Aug 12;20(8):e0328521. doi: 10.1371/journal.pone.0328521 (PMC12342280; doi:10.1371/journal.pone.0328521)

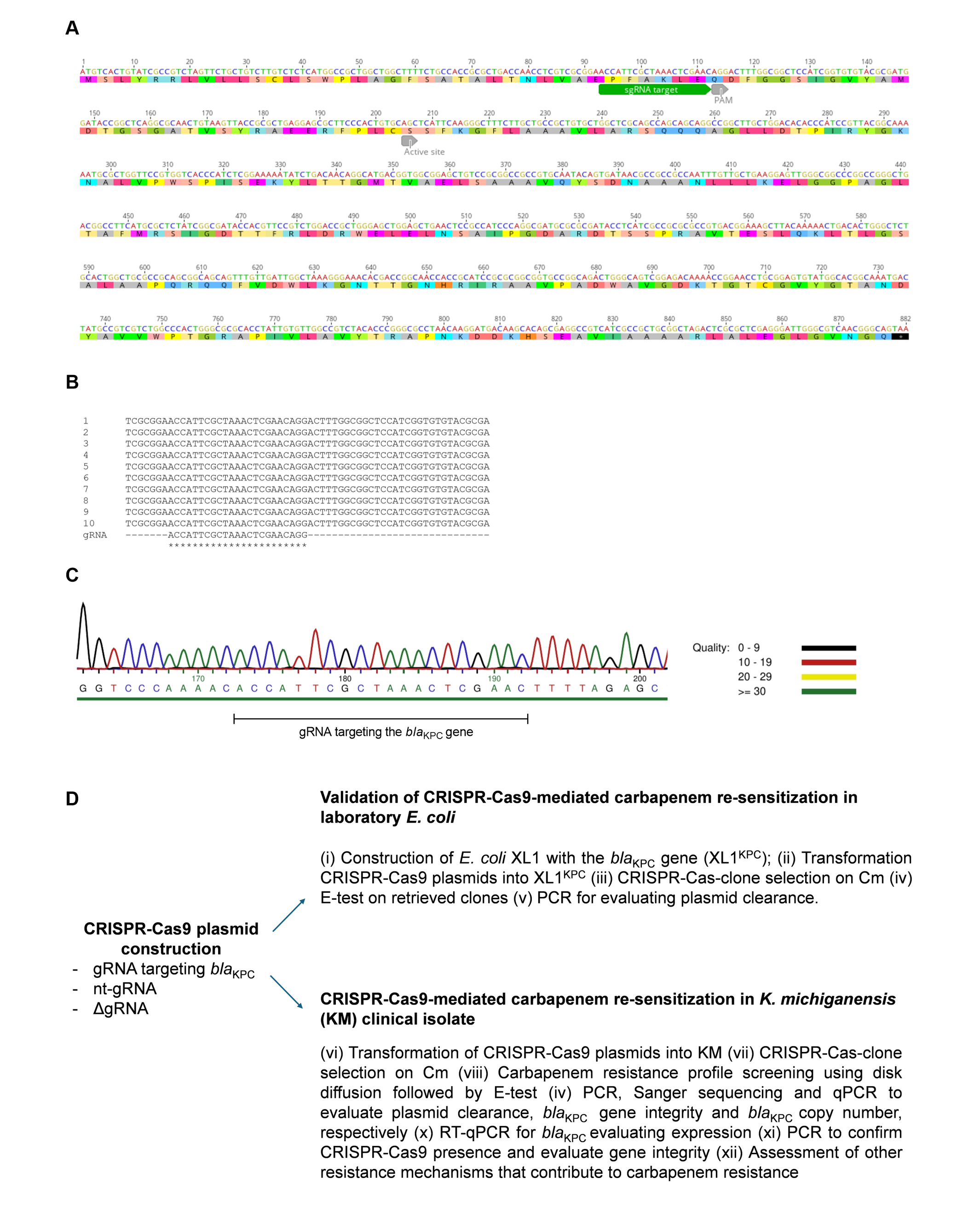

Supplement: S1 Fig — S1A. Full blaKPC gene sequence and the position of the designed gRNA at the 5’ end, before the region encoding for the active site of the KPC S1B. Alignment of representative blaKPC variants from Genbank showing that the designed gRNA targets a conserved region. The selected genes and sequence accession number are: 1. blaKPC-2 KT001101.1; 2. blaKPC-25 KU216748.1; 3. blaKPC-2 KT001098.1; 4. blaKPC-2 KT001097.1; 5. blaKPC-19 KJ775801.1; 6. blaKPC-3 AF395881.1; 7. blaKPC-1 AF297554.1; 8. blaKPC-12 HQ641421.1; 9. blaKPC-2 GU086225.1; 10 blaKPC-14 JX524191.1. S1C. Sequence fragment of the CRISPR-Cas9 plasmid showing correct insertion of the gRNA. Green line below the assigned nucleotides indicates sequencing quality, according to the colour code legend. S1D. Schematic workflow of the experimental study design. Briefly, the three CRISPR-Cas9 plasmids used in this study were first constructed. CRISPR-Cas9 mediated re-sensitization ability was validated in a laboratory strain of E. coli. Following successful re-sensitization, the CRISPR-Cas9 effect was evaluated on a clinical strain of K. michiganensis. (TIF) [file pone.0328521.s001.tif]

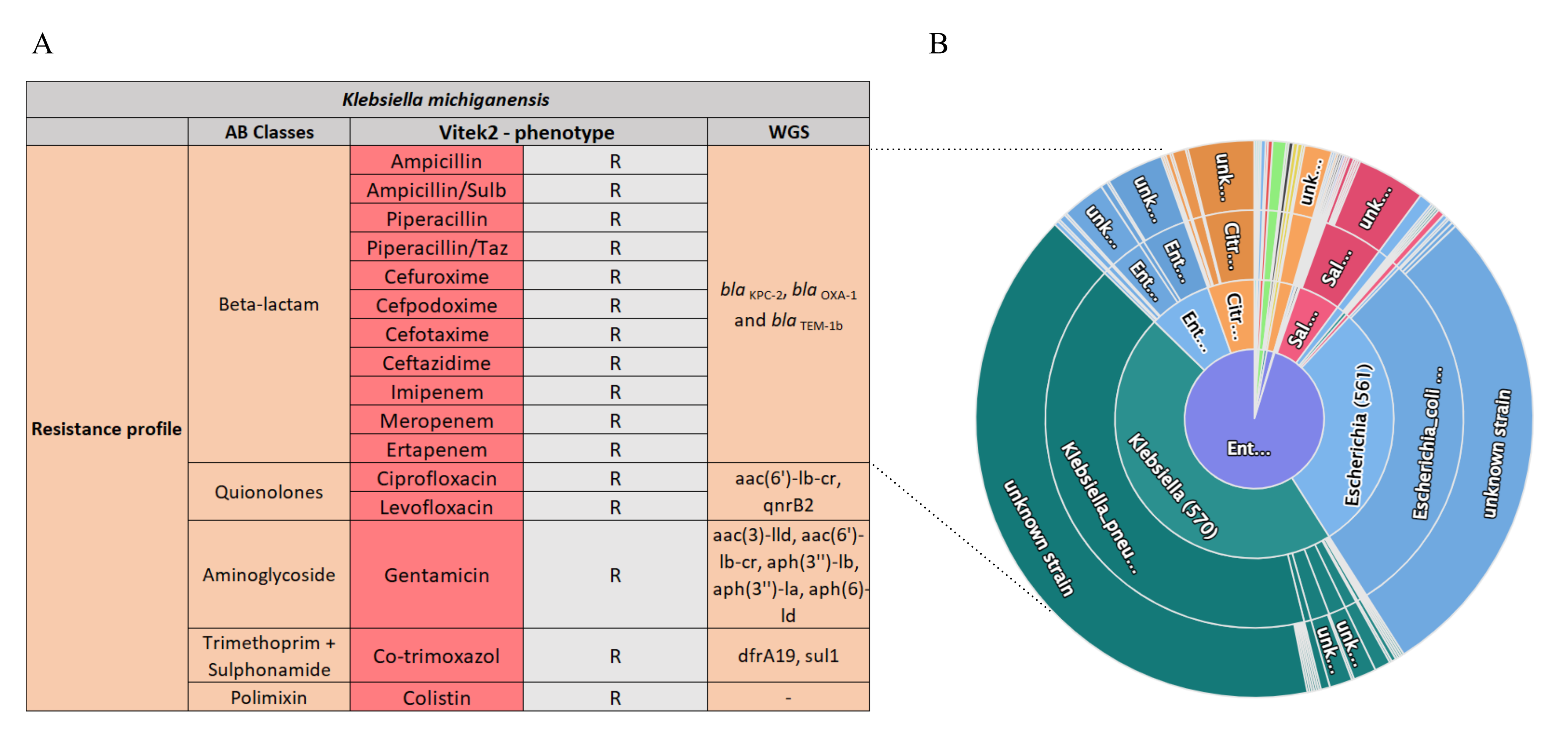

Supplement: S2 Fig — S2A. Resistance profile of K. michiganensis KM and equivalent resistance genes determinants identified by WGS. S2B. The blaKPC gene is carried by an IncN plasmid, which has nucleotide similarity with plasmids mostly harboured by Enterobacteriaceae, especially Klebsiella and Escherichia coli. PLSDB database graphic: Centred circle “Ent…”: Enterobacteriaceae; “Ent…”: Enterobacter; “Citr…”: Citrobacter; “Sal…”: Salmonella; “unk”: unknown. (TIF) [file pone.0328521.s002.tif]
