# Supplementary material for: CRISPR-Cas9 targeting the blaKPC gene in a clinical isolate of Klebsiella michiganensis: Reduction of imipenem resistance and changes in genomic carbapenem resistance determinants
Source: PLoS One. 2025 Aug 12;20(8):e0328521. doi: 10.1371/journal.pone.0328521 (PMC12342280; doi:10.1371/journal.pone.0328521)
Supplement: S3 Fig — (PDF) [file pone.0328521.s003.pdf]

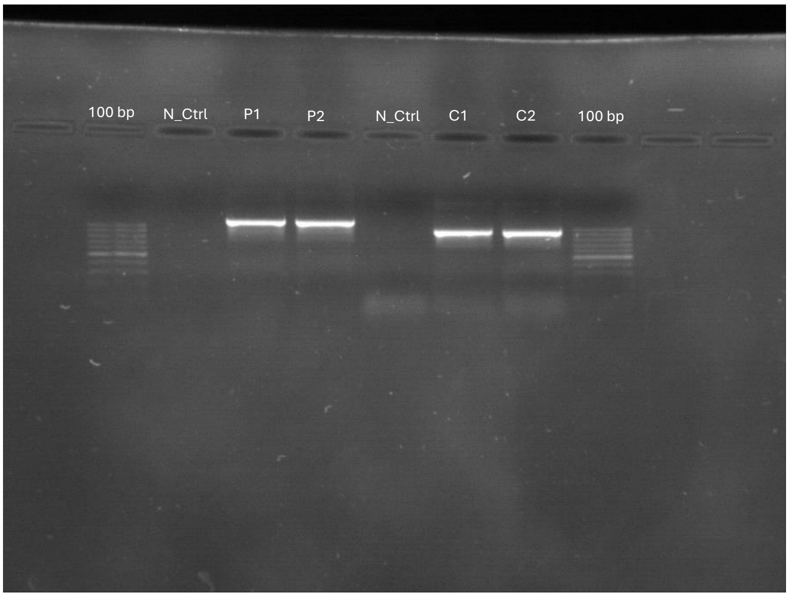

Fig S3A. Agarose gel showing PCR amplified *bla*$_{KPC}$ genes from two different PCR set ups: samples P1 and P2 shows amplified fragment of the *bla*$_{KPC}$ region and its putative natural promoter; samples C1 and C3 shows amplified fragment of the *bla*$_{KPC}$ region with a synthetic promoter located on the primer, i.e. forward primer containing the Anderson promoter J23101 and RBS regions at the 5'-end. Clones C1 and C2 were selected for this study and the corresponding gel picture added in Figure 1A. N_Ctrl: PCR negative control.

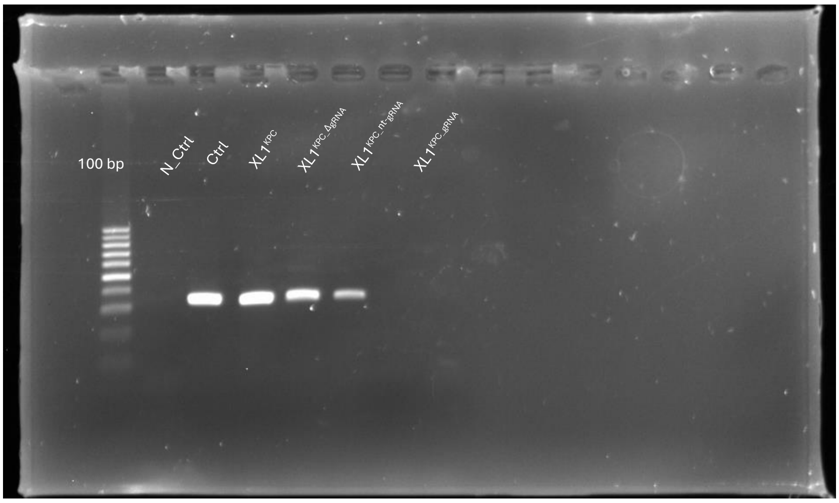

Fig S3B. Agarose gel showing successful PCR amplification of an inner fragment of the *bla*$_{KPC}$ gene except for XL1^{KPC_gRNA}, suggesting effective re-sensitization due to *bla*$_{KPC}$ plasmid clearance. N_Ctrl: PCR negative control; Ctrl: PCR positive control. XL1: *E. coli* lacking the *bla*$_{KPC}$ gene; XL1^{KPC}: XL1 transformed with the *bla*$_{KPC}$ gene; XL1$^{KPC_\Delta gRNA}$: XL1^{KPC} treated with CRISPR-Cas9 without gRNA; XL1$^{KPC_nt\text{-}gRNA}$: XL1^{KPC} treated with CRISPR-Cas9 with the non-targeting gRNA; XL1^{KPC_gRNA}: a XL1^{KPC} treated with CRISPR-Cas9 with the targeting gRNA. Picture was adjusted and added in Figure 1C.

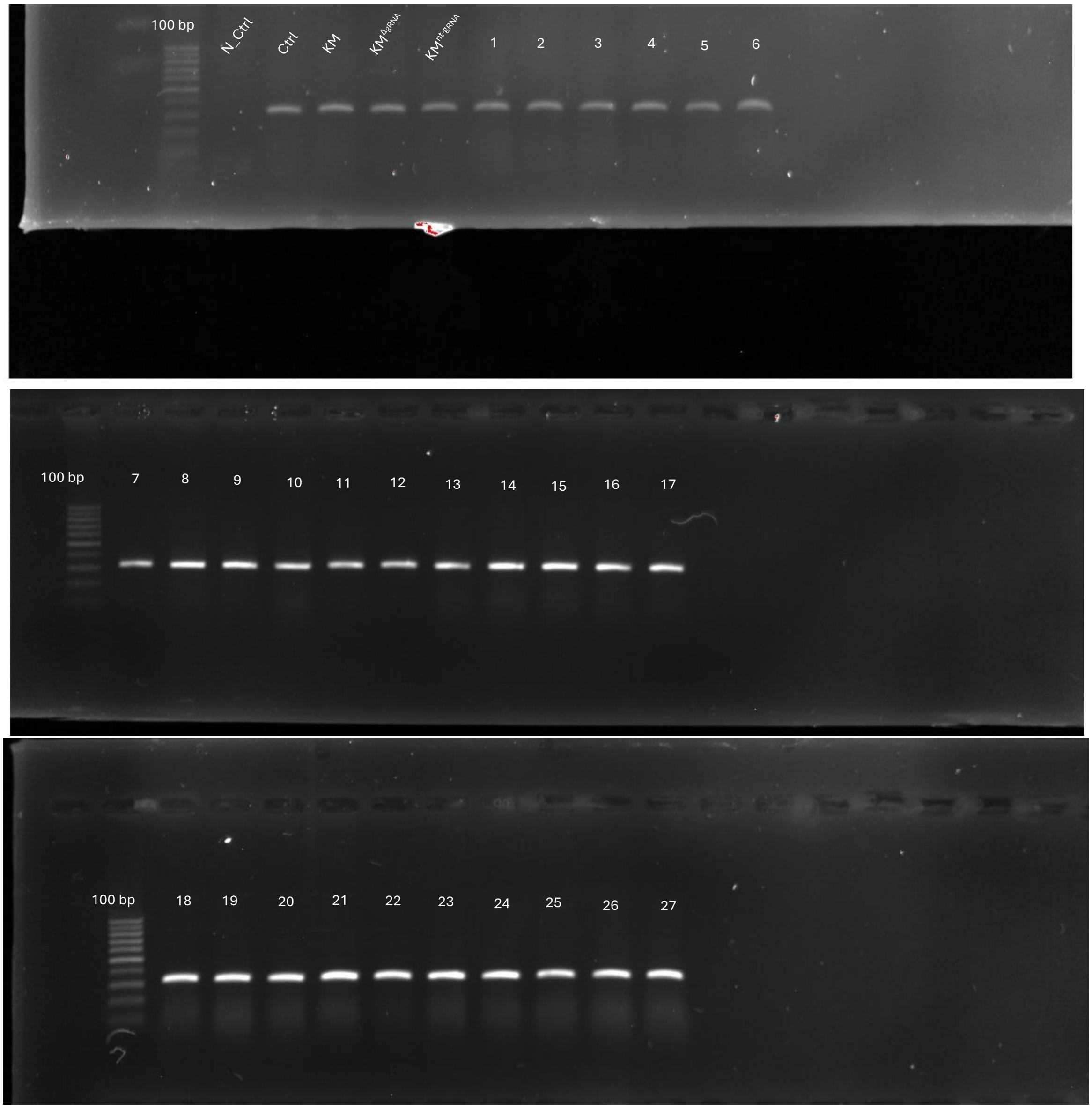

Fig S3C. Three different agarose gel showing PCR amplification of a *bla*$_{KPC}$ gene fragment in all transformants indicating that re-sensitization was not due to resistance plasmid clearance. N_Ctrl: PCR negative control; Ctrl: PCR positive control; KM$^{\Delta gRNA}$: KM treated with CRISPR-Cas9 without gRNA; KM$^{nt\text{-}gRNA}$: KM treated with CRISPR-Cas9 with a non-targeting gRNA; 1-27: clone numbers of KM treated with CRISPR-Cas9 with the targeting gRNA. Gel pictures were adjusted and added in Figure 3A.

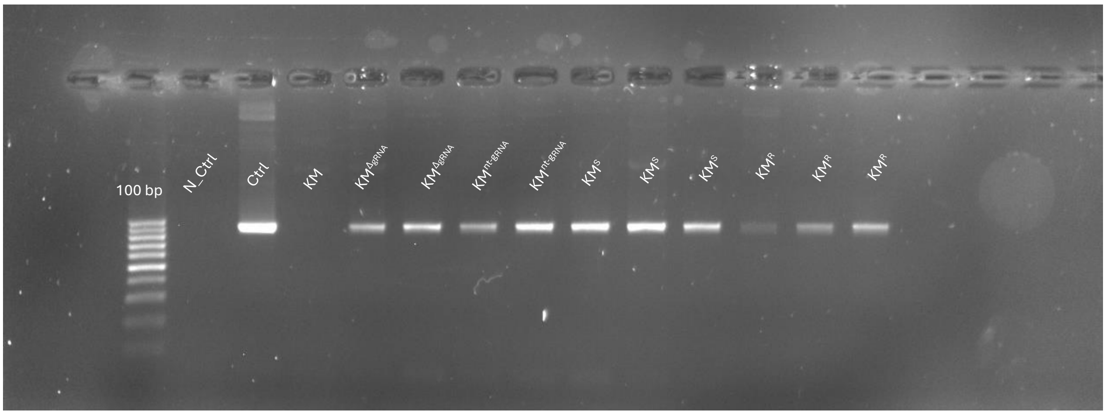

Fig S3D. Agarose gel showing PCR amplification of a fragment of the CRISPR-Cas9 carrying vector indicating its successful transformation in all assessed transformants. N_Ctrl: PCR negative control; Ctrl: PCR positive control; KM$^{\Delta gRNA}$: KM treated with CRISPR-Cas9 without gRNA; KM$^{nt\text{-}gRNA}$: KM treated with CRISPR-Cas9 with a non-targeting gRNA; KM^{S}: imipenem re-sensitized transformants of KM^{gRNA}; KM^{R}: transformants of KM^{gRNA} with continued imipenem resistance. Picture was adjusted and added in Figure 3E.

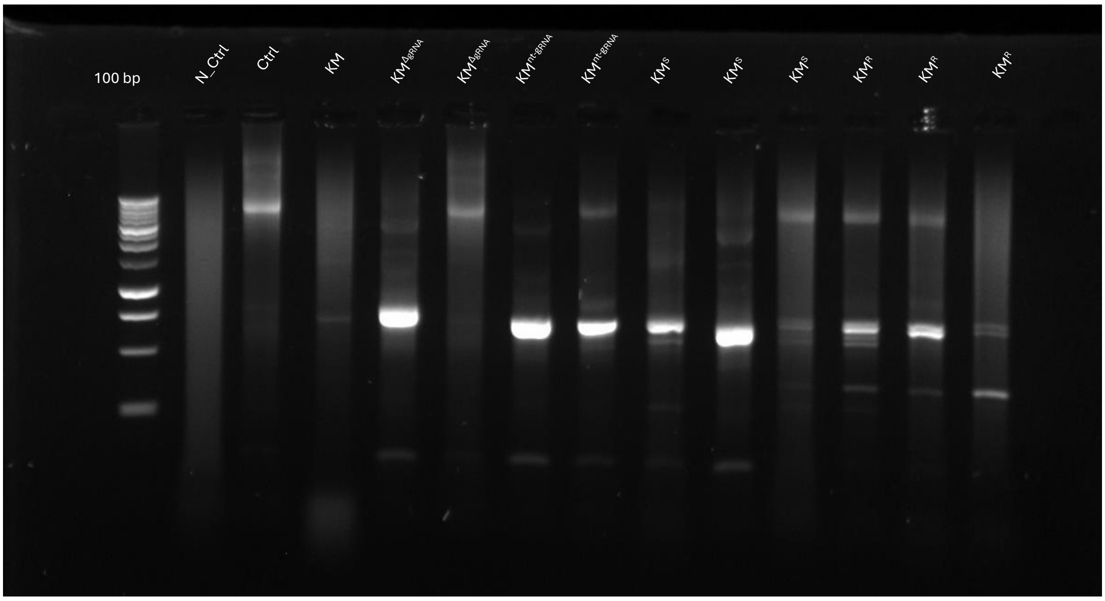

Fig S3E. Agarose gel showing PCR amplification of the entire CRISPR-Cas9 system (~ 5.4 kb), indicating compromised integrity in representative transformants. N_Ctrl: PCR negative control; Ctrl: PCR positive control; KM$^{\Delta gRNA}$: KM treated with CRISPR-Cas9 without gRNA; KM$^{nt\text{-}gRNA}$: KM treated with CRISPR-Cas9 with a non-targeting gRNA; KM^{S}: imipenem re-sensitized transformants of KM^{gRNA}; KM^{R}: transformants of KM^{gRNA} with continued imipenem resistance. Picture was adjusted and added in Figure 3F.
